# Statistical complexity of reasons for encounter in high users of out of hours primary care: analysis of a national service

**DOI:** 10.1186/s12913-019-3938-z

**Published:** 2019-02-08

**Authors:** Sarah Stegink, Alison M. Elliott, Christopher Burton

**Affiliations:** 1 Medical School, University of Aberdeen, Aberdeen, UK; 2 Abertay University, Dundee, UK; 3 Institute for Applied Health Sciences, University of Aberdeen, Aberdeen, UK; 4 Academic Unit of Primary Medical Care, University of Sheffield, Samuel Fox House, Northern General Hospital, Sheffield, S5 7AU UK

**Keywords:** Complexity, Entropy, Frequent attendance, High users, Out of hours care, Reason for encounter, Unscheduled care

## Abstract

**Background:**

Managing demand for urgent and unscheduled care is a major problem for health services globally. A particular issue is that some patients appear to make heavy use of services, including primary care out of hours. We hypothesised that greater variation (statistical complexity) in reasons for attending primary care out of hours services may be a useful marker of patients at high risk of ongoing heavy service use.

**Methods:**

We analysed an anonymised dataset of contacts with the primary care out of hours care for Scotland in 2011. This contained 120,395 contacts from 13,981 high-using patients who made 5 or more contacts during a calendar year. We allocated the stated reason for each encounter into one of 14 categories. For each patient we calculated measures of statistical complexity of reasons for encounter including the count of different categories, Herfindahl index and statistical entropy of either the categories themselves, or the category transitions. We examined the association of these measures of statistical complexity with patient and healthcare use characteristics.

**Results:**

The high users comprised 2.4% of adults using the service and accounted for 15% of all contacts. Statistical complexity (as entropy of categories) increased with number of contacts but was not substantially influenced by either patient age or sex. This lack of association with age was unexpected as with increasing multi-morbidity one would expect greater variability in reason for encounter. Between 5 and 10 consultations, higher entropy was associated with a reduced likelihood of further consultations. In contrast, the occurrence of one or more contacts for a mental health problem was associated with increased likelihood of further consultations.

**Conclusion:**

Complexity of reason for encounter can be estimated in an out of hours primary care setting. Similar levels of statistical complexity are seen in younger and older adults (suggesting that it is more to do with consultation behaviour than morbidity) but it is not a predictor of ongoing high use of urgent care.

**Electronic supplementary material:**

The online version of this article (10.1186/s12913-019-3938-z) contains supplementary material, which is available to authorized users.

## Background

Managing demand for urgent and unscheduled care is a major problem for health services globally. A particular issue is that some people make heavy use of urgent and unscheduled care both in hospital settings [1, 2] and in primary care [3–5]. There are strong similarities in statistical patterns of attendance across these two apparently different settings [6]. Many high users have complex medical and care needs [4, 7–9], This complexity may include multiple physical diseases, mental disorders [7, 10] (including health anxiety and substance misuse disorders [11]) and conditions which include both physical and mental components such as persistent physical symptom disorders (also known as “medically unexplained symptoms” or somatoform disorders) [5]. We hypothesised that greater variation in reasons for attending urgent and unscheduled care could be analysed in terms of statistical complexity and might be a useful marker of patients at high risk of ongoing heavy service use.

At present there are no widely used ways of measuring statistical complexity in routinely collected urgent and unscheduled care data. While statistical measures of complexity have been developed for consultations [12] based on the nature of tasks involved and for cases based on co-morbid conditions [13], we are not aware of measures which look at complexity of reason for encounter across multiple episodes of illness or treatment. Based on approaches in information science or ecology, statistical approaches to complexity across multiple illness episodes ought to describe the unpredictability or diversity of reasons for attendance. From this perspective, for a given number of attendances, a more complex case will have more varied reasons for encounter: in contrast a simpler case will have less varied reasons for encounter. This statistical complexity might arise either from multi-morbidity (more different diseases leading to consultation) or from generally increased help seeking (a greater tendency to consult for conditions which might not otherwise lead to urgent care contact). The closest equivalent research relates to studies of continuity of care [14–16], where low continuity of care is equivalent to high statistical complexity.

We aimed to estimate the statistical complexity of patients’ reasons for encounter in a large routinely collected dataset of primary care out of hours consultations using a range of different measures. We then aimed to compare these measures and examine their associations with patient characteristics including age, sex, and whether the reasons for encounter included a mental health problem or particular patterns of physical symptoms. Finally, we aimed to examine the predictive value of complexity measures in identifying individuals with a given number of contacts who then went on to have further contacts.

## Methods

### Data source

We examined a large routinely-collected database of anonymised urgent care use [17, 18] comprising all contacts over one calendar year with NHS24, the primary care out-of-hours (PCOOH) service for the whole of Scotland, United Kingdom (population 5 M). NHS24 provides a range of primary care services, mostly when general practices are closed (typically between 18.00 and 08.00 plus weekends and public holidays) including telephone triage and advice, face to face consultation at designated hubs and home visits by a member of the PCOOH team. It is used by almost all general practices in Scotland. A more detailed description analysis of the service has been published elsewhere [17]. Each contact by a patient with the service was logged (date, time and reason for encounter) and linked to a unique patient identifier. Contacts were allocated a reason for encounter (RfE) by the call-handler from a menu.

The database included all calls made to NHS24 in 2011. For the analysis, we limited the dataset to adults over the age of 18 and to calls made during the out of hours period when normal GP services were closed. While each contact was attributable to a specific patient, the data available did not include patient-identifying information: the only demographic data was the patient age and sex.

### Categories of reason for consultation

To provide a manageable number of categories for RfE we mapped all the codes used by call-handlers to one of fourteen categories. These were based on symptoms reported by the patients rather than the ultimate diagnoses. Categories were chosen to cluster together broadly similar items. We used four specific groups of physical symptoms (musculo-skeletal, cardio-respiratory, gastro-intestinal and general / neurological) to map to the body systems used in the bodily distress syndrome (BDS) [19, 20]. We designated these as BDS systems and calculated a score per patient of the number of these systems with at least one RfE. We included these because the presence of BDS features may indicate a more systematic disorder of heightened symptom processing and high healthcare use [21] and because similar disorders have been associated with frequent PCOOH attendance [5]. We included a major illness category for calls specifically about conditions such as cancer or diabetes; however symptomatic episodes of illness such as exacerbations of chronic lung diseases were typically included under their presenting symptom (e.g. cough or breathlessness). The final categorisation was mental health. For the primary analysis we placed calls relating to alcohol and substance misuse in the “other” category, however for a secondary analysis we included them with mental health problems. The full mapping of call-handler codes to symptom categories is shown in Additional file 1: Data 1.

### Minimisation of duplicate data

The database contained some instances of multiple calls per episode of care. This could arise, for instance, when a patient called back because a symptom was changing or to confirm that someone was on the way to assess them. For the analysis, we excluded repeated contacts which we defined as relating to the same category and on the same day as another contact.

### Threshold for designating “high-use”

We set a threshold for high use of 5 or more contacts in the calendar year. We chose this as representing the best trade-off between a large enough number of contacts to examine heterogeneity and the number of patients who would be included. For the analysis we created four sub-groups based on total number of contacts in the year: 5–10, 11–20, 21–30 and > 30.

### Calculation of complexity

We took the idea of measuring complexity from methods developed in information theory and widely used in sciences such as ecology [22]. These methods produce a statistic which represents the amount of information required to describe a feature (whether a sequence of consultations or an ecological habitat).

We calculated four measures of complexity in RfE per patient. First, we used the count of different categories of RfE per patient. While this is easy to estimate, it cannot differentiate between the proportion of contacts which occur in each of the categories represented. Second, we calculated the Herfindahl index, which is an economic tool for measuring market share and represents the sum of the squares of proportion of consultations for each RfE. It is closely related to the Bice-Boxerman index but has the mathematical merit of always scaling between zero and one. Both the Herfindahl and Bice-Boxerman indices have been used in measurement of continuity of care [14, 23]. Third, we estimated Shannon entropy of the proportion of consultations for each RfE: this is a more sophisticated measure of diversity derived from information theory, and is used extensively, for instance in ecology to describe diversity of species in a habitat [22]. We refer to this measure as state entropy because it describes the complexity of the different states, or categories, of RfE but not their sequence. Finally, we estimated Shannon entropy of the transitions between one RfE and the next which we refer to as transition entropy. Formulae for these measures are listed in Additional file 2: Data 2. Table 1 illustrates these measures, using the hypothetical example of four different sequences of 8 contacts for three possible reasons (A, B and C) with calculated values for each of the complexity measures. The count of RfE is unable to differentiate between any of the bottom three rows. While state entropy is able to discriminate between sequences with different proportions of the three RfEs, only transition entropy is able to differentiate between all sequences.

**Table 1:** example of different complexity measures from an example sequence of reasons for consultation (RfE)

	Count (RfE)	Herfindahl Index^a^	State Entropy	Transition Entropy
AAABAAAB	2	0.78	0.5	1.1
AAABAAAC	3	0.59	1.1	1.7
AAABBBCC	3	0.34	1.6	2.2
AABCCABB^b^	3	0.34	1.6	2.8

^a^Formulae for calculating Herfindahl Index and State and Transition Entropies are in Additional file 2: Data 2

^b^Note the bottom two rows are equivalent for Herfindahl index and state entropy (both contain 3A’s 3B’s and 2C’s. For the transition entropy the bottom row contains only one repeated transition (AB), the others all occur only once (AA, BC,CC,CA, BB).

### Statistical methods

Complexity measures were estimated using standard formulae implemented in R 3.4.2. We compared the four complexity measures in three ways. First, we plotted histograms of the distribution of values in each of the four subgroups representing different levels of use over the 12 months. Second, we considered the number of contacts needed to reach a relatively stable value by taking a random sample of very high users (over 30 contacts) and plotting the value of the measure over the first *N* RfEs where *N* ranged from 5 to 30. Third, we examined the relationship between measures by creating scatterplots and by calculating correlations.

We tested associations of complexity measures with patient demographics and mental health by generating box-plots and by using simple and multiple linear regression. Analysis was carried out on a dataset including all patients. However, to exclude the possibility of our findings being heavily influenced by a few extremely high users, we repeated the analysis, limiting it to patients with between 5 and 30 contacts in the year. We included variables in the multiple regression model if the univariate regression coefficient had a *p*-value < 0.1 and where testing for variance inflation factor showed low multi-collinearity. We assessed the appropriateness of multiple linear regressions by plotting residuals against a normal distribution.

Finally, we examined whether measures of complexity, estimated after a given number of consultations, were associated with further consultation. We analysed data for patients with at least N_1_ consultations (where N_1_ varied between 5 and 15) and used measures of complexity from their first N_1_ consultations to predict whether they would have N_2_ consultations (where N_2_ was either N_1_ + 1 or N_1_*1.333). We used logistic regression to examine the effect on further consultation of the following predictors: (a) complexity (as transition entropy of the first N_1_ contacts, standardised for ease of interpretation) (b) the presence of any mental health RfEs in the first N_1_ contacts (c) the number of unique RfEs in the first N_1_ contacts.

### Consent and other permissions

All data was anonymised and handled under a data-sharing agreement between the University of Aberdeen and NHS24; as no patient identifiable data was involved, additional research ethical permission was not required.

## Results

The database contained 947,814 contacts involving 577,324 adult patients. Of these we excluded 151,995 contacts (95,279 occurred “in hours” rather than during the out of hours period, 38,419 had no problem code entered by the call-handler and 18,297 were related to pregnancy and childbirth). We excluded a further 13,556 duplicate contacts (in the primary analysis, numbers varied in the sensitivity analysis) leaving 782,281 contacts by 507,934 patients. Of these contacts, 120,395 (15.1%) occurred in the 13,981 (2.4%) patients who made 5 or more out of hours contacts.

The high using patients included 8852 women (63.3%) and 5129 men (36.7%). The proportions of female and male high-users was similar to the proportion of women and men among adults who made any contact with the service (62 and 38% respectively). 2635 (18.8%) were aged 18–30; 3185 (22.8%) aged 31–50; 3026 (21.6%) aged 51–70; and 5135 (36.7%) aged over 70. 2708 (19.4%) high-using patients had at least one contact for a mental health problem and 1176 patients (8.4%) had at least one contact specifically about a major illness such as cancer or diabetes. Table 2 shows the distribution of reasons for encounter in both low users and the high users, with the difference in the incidence of RfE categories between the high users and the remaining patients presented as odds ratios. Contacts with high users were relatively more likely to be for mental health reasons (OR = 3.26) and less likely to be for minor conditions such as upper respiratory infections (OR = 0.43) or Skin, Eye and Ear Nose & Throat (ENT) (OR = 0.33).

**Table 2:** Number (and proportion) of contacts by each reason for encounter in high and low users

RfE Categories		High Users		Low Users		Odds ratio	95% CI
Description	N	Count	%	Count	%		
Accident	29,379	3630	3.2	25,749	3.8	0.83	0.80 to 0.86
Musculoskeletal^a^	102,019	11,912	10.4	90,107	13.2	0.76	0.74 to 0.78
Gastrointestinal^a^	120,022	17,651	15.4	102,371	15	1.03	1.01 to 1.05
Neuro &General^a^	69,276	8898	7.8	60,378	8.9	0.86	0.84 to 0.88
Cardiopulmonary^a^	94,698	14,308	12.5	80,390	11.8	1.06	1.04 to 1.08
Mental Health	24,732	8479	7.4	16,253	2.4	3.26	3.17 to 3.35
Major conditions	10,489	1847	1.6	8642	1.3	1.27	1.21 to 1.34
Reproductive	62,317	13,175	11.5	49,142	7.2	1.67	1.64 to 1.70
Skin Eye ENT	114,876	6557	5.7	108,319	15.9	0.32	0.31 to 0.33
Upper Respiratory	27,580	1915	1.7	25,665	3.8	0.43	0.41 to 0.45
Other	727	160	0.1	567	0.1	1.68	1.41 to 2.00
Medication	33,541	6398	5.6	27,143	4	1.42	1.38 to 1.46
Safety	85,533	15,124	13.2	70,409	10.3	1.32	1.30 to 1.35
Uncategorised	20,648	4729	4.1	15,919	2.3	1.8	1.74 to 1.86
Total	795,837	114,783		681,054			

*RfE* Reason for Encounter, *ENT* Ear Nose and Throat

^a^Categories included in the bodily distress syndrome systems

### Comparison of complexity measures

**Fig. 1:**
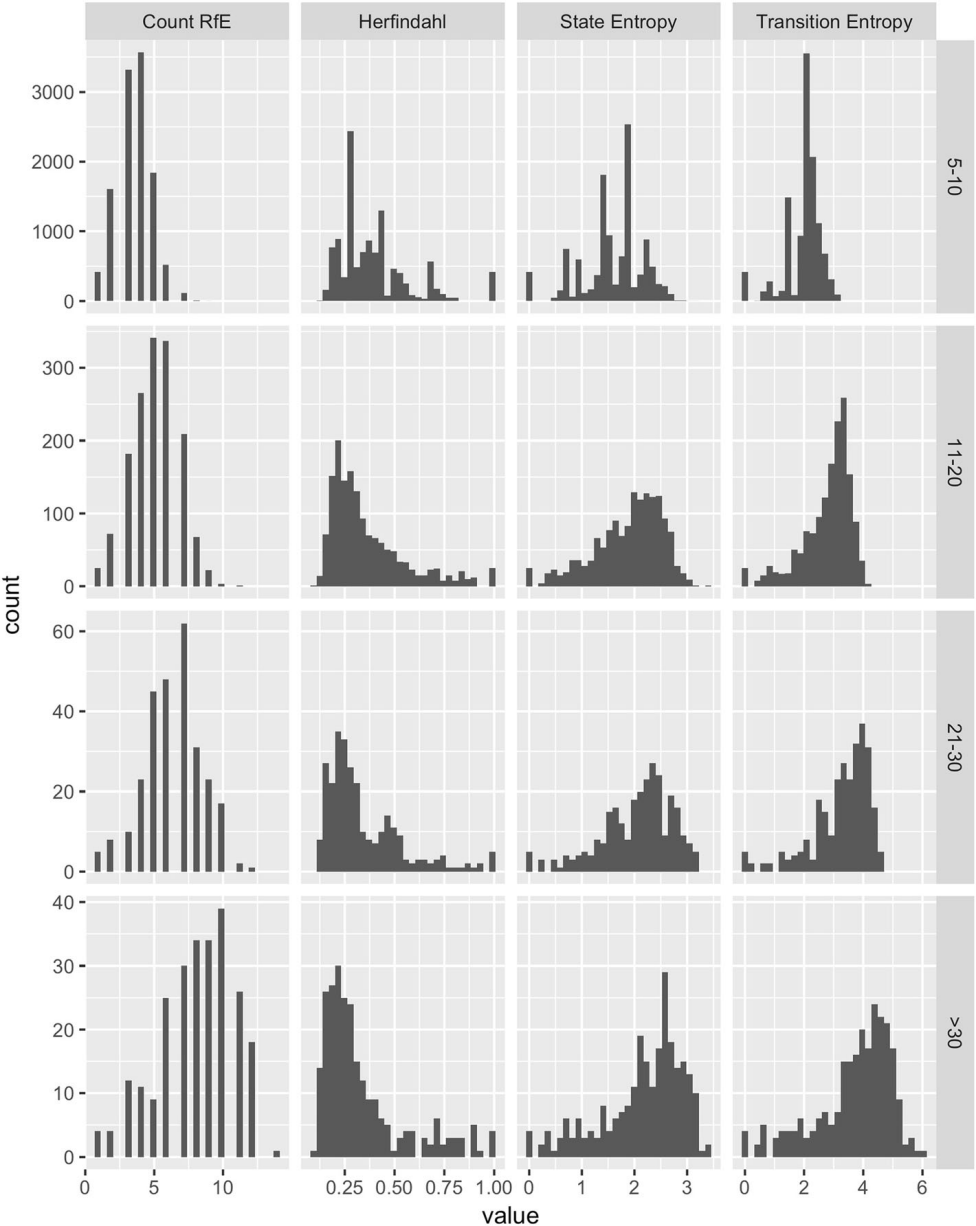
Distributions of each of the four complexity measures

The distributions of each of the four complexity measures are shown in Fig. 1. Median value (with interquartile range) for the count of RfE categories was 4 (3 to 5); for state entropy it was 1.75 (1.37 to 2) and for transition entropy was 2.0 (1.9 to 2.5). All four measures were closely correlated (allowing for the fact that Herfindahl index is scaled in the opposite direction to the other three measures: high complexity is associated with a lower value) and details of this are shown in Additional file 3: Figure S1. The number of contacts to achieve a relatively stable value is shown in Additional file: 4 Figure S2 – of the four measures, state entropy appears to be the most stable over increasing numbers of contacts.

Based on the information in Fig. 1 and Additional file 3: Figure S1 and Additional file 4: Figure S2, we chose state entropy as the measure of complexity for inclusion in the subsequent analysis.

### Relationship of complexity to patient characteristics

**Fig. 2:**
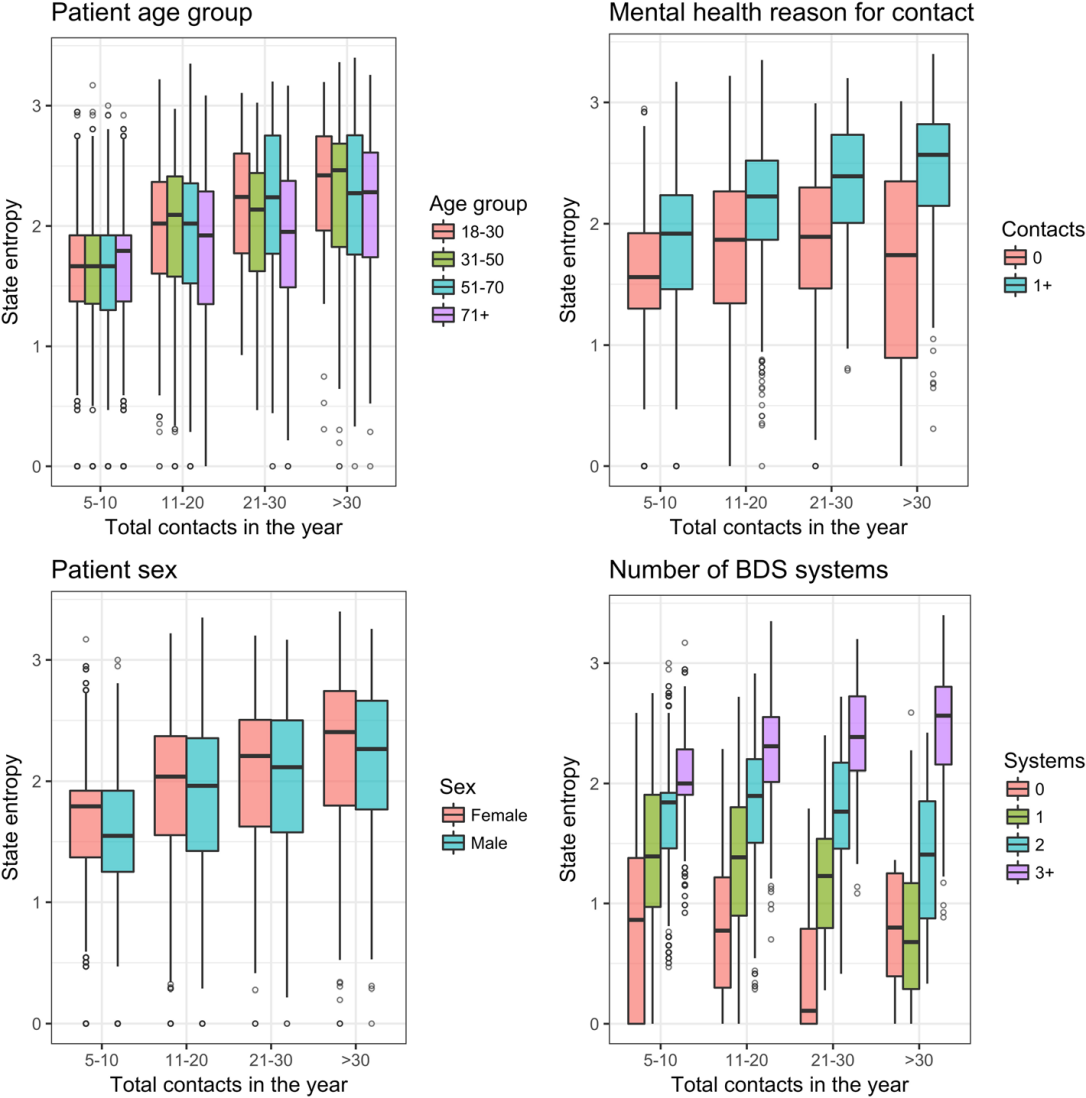
Box plots of State Entropy by patient age group, sex, presence of any mental health reason for encounter and by number of bodily distress syndrome categories.BDS: Bodily Distress Syndrome

Figure 2 shows the relationship of complexity (as state entropy) to patient age group; sex; presence of a mental health contact; and number of BDS systems. In each plot, the complexity increases with number of contacts. Differences in complexity between people with and without a mental health RfE only become apparent in those patients with more than 10 contacts.

Table 3 shows the result of univariable regressions of each of the variables as predictors of state entropy. In this analysis, each variable apart from age has a significant association with state entropy. The second and third sections of Table 3 show the coefficients of the multiple linear regression for all high users and then for the subset of patients who had between and 5 and 30 contacts. This shows that the effects of mental health RfE and number of BDS system categories on state entropy were absent or weak after adjusting for other consultation pattern features. Additional file 5: Data 3 includes the histograms of residuals from the two multiple regression models. While these do not fit exactly to a normal distribution, the plot for the subset of patients is relatively symmetrical. Both multiple regression models accounted for a large proportion of the variance in Shannon entropy: adjusted-R^2^ for the model with all patients was 0.86 and for the patients with < 30 contacts it was 0.92.

**Table 3:** Simple and multiple regression coefficients for variables associated with State Entropy

	Coefficient	95%CI	*p*-value
Simple regression – all high users			
Number of calls	0.01	0.010 to 0.010	<.001
Number of RfE categories	0.35	0.350 to 0.350	<.001
Number of BDS systems	0.51	0.490 to 0.520	<.001
Age (decades)	0.00	−0.004 to 0.004	0.86
Sex: Male	−0.09	−0.110 to −0.070	<.001
Mental Health RfE	0.34	0.310 to 0.370	<.001
Multiple regression - all high users			
Number of calls	−0.017	−0.018 to − 0.017	<.001
Number of RfE categories	0.406	0.403 to 0.408	<.001
Sex: Male	−0.021	−0.029 to − 0.013	<.001
Mental Health RfE	−0.046	− 0.056 to − 0.036	< 0.001
Multiple regression - users with 5–30 contacts			
Number of calls	− 0.047	−0.048 to − 0.046	<.001
Number of RfE categories	0.452	0.450 to 0.454	<.001
Sex: Male	− 0.014	− 0.02 to − 0.008	< 0.001
Mental Health RfE	0.046	0.056 to 0.036	< 0.001

*RfE* Reason for encounter, *BDS* Bodily distress syndrome

Age excluded from the multiple regression as no significant effect in univariate regression. BDS excluded from multiple regression because of multicollearity.

### Predictive value of complexity of RfE on future contact

**Fig. 3:**
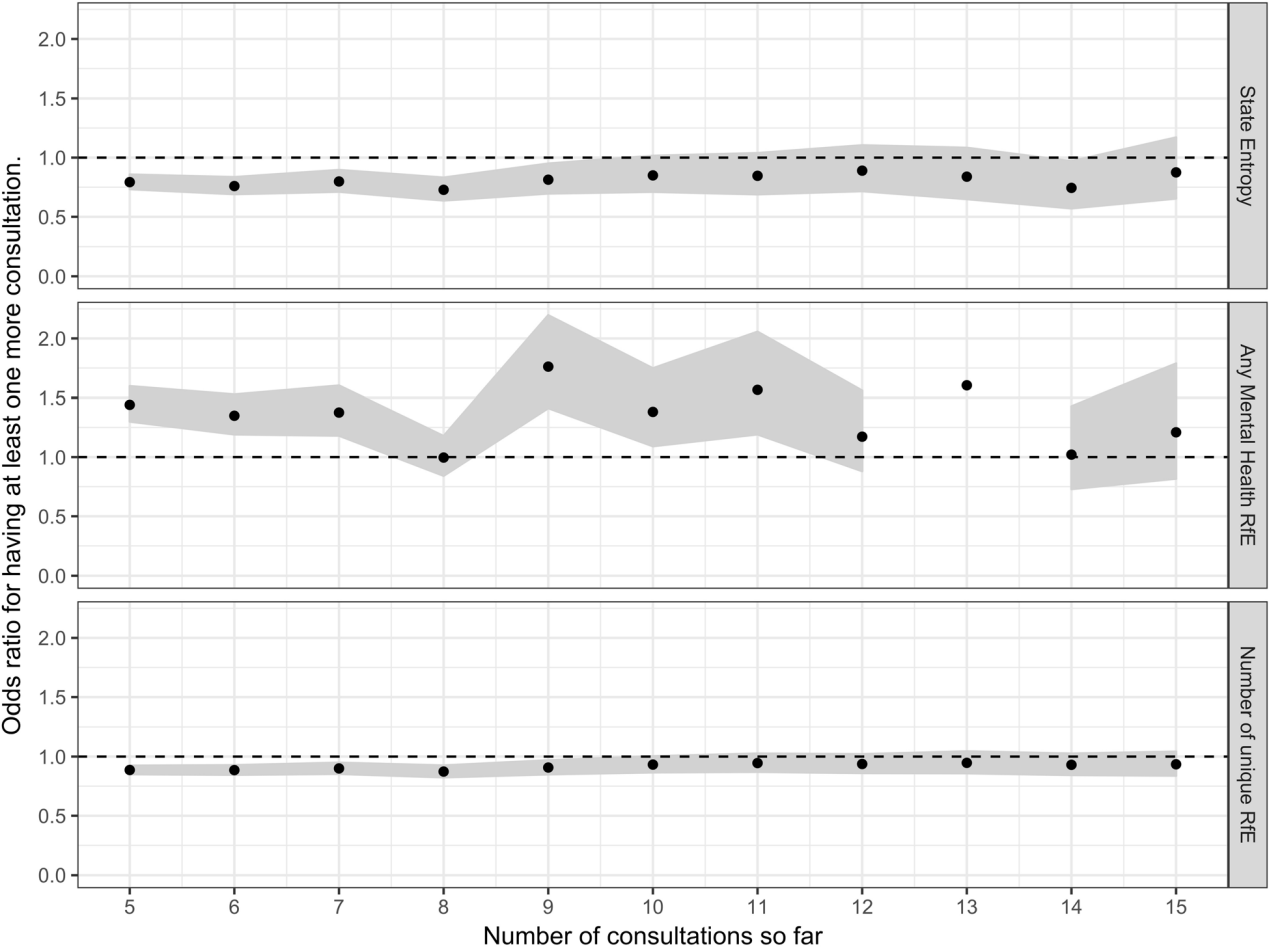
Effect of stated features on the probability of one or more further contacts after a given number of contacts (as odds ratio)

Figure 3 shows the influence of selected features (number of RfE, state entropy and having had one or more mental health RfE), on the probability of having one or more additional consultations for each given number of consultations so far. Results are shown as odds ratios with 95% confidence intervals and represent the results of logistic regression, adjusted for age and sex, and limited to patients with at least 2 different RfE in the total period. The figure shows that while the presence of any prior mental health consult is modestly predictive of further consultation (odds ratio between 1.2 and 1.9) at any number of contacts above 5, state entropy is associated with a lower likelihood of further consultations between 5 and 10 contacts. While Fig. 3 shows the effect of predictors on one more consultation, similar patterns are seen when these features are used to predict 33% more consultations (Additional file 6: Figure S3).

## Discussion

### Summary of main findings

This is the first study to our knowledge to calculate and describe statistical measures of complexity of reasons for encounter with health services. Two measures appeared promising: count of contact categories (which is simple to calculate) and Shannon entropy of RfE categories (which is more difficult to calculate but has better statistical properties). Patient age had only small effects on statistical complexity of reason for encounter suggesting that statistical complexity of urgent care use is more related to healthcare seeking behaviour than to underlying multi-morbidity. Statistical complexity of reason for encounter was not predictive of future consultation, although having attended for a mental health problem was.

### Strengths and limitations

This study used a very large and recent dataset of out of hours primary care use which has been validated and analysed in earlier publications [17, 18]. The analysis used a range of techniques for estimating complexity, and the categorisation of physical symptoms followed existing patterns [24]. By considering a range of metrics we were able to choose between them on the basis of their statistical properties and by examining the predictive value of statistical complexity measures for further contact across a range of consultation numbers, we ensured that the results were not sensitive to particular parameters.

The use of patient-initiated reason for consultation was both a strength and a limitation. Its strength was that it reflected patients’ experience of what they perceived to be the problem (or perhaps an appropriate lever to access help). The use of reason for encounter has also been advocated through its inclusion in the International Classification of Primary Care (ICPC-2) [25]. Its limitation is that it made it impossible to distinguish calls due to new incident symptoms from exacerbations of existing conditions in a way that a diagnosis based classification might. This was particularly limiting in the case of mental health conditions as we were only able to identify patients in whom mental health was a reason for encounter, not those in whom it may have been a comorbidity (such as depression) or a contributing factor (such as health anxiety [26] or accompanying a somatoform disorder [5]).

We were limited to only using contacts for unscheduled care during the out-of-hours period because of the data. However, an increasing tendency within UK general practice to facilitate same-day access for all problems would mean that even if such data were available it could not be interpreted. Furthermore we found very similar distributions of contacts per patient between this dataset and 17 other datasets from a range of urgent and unscheduled care settings [6]. The dataset was limited to one calendar year – this was part of the original specification and could not be changed. However, one year is a typical period for studies of attendance.

The analysis posed challenges because of the non-normal distributions of variables – particularly number of contacts [6] and number of different RfE (which were also correlated). Additional file 7: Data 4 describes an additional analysis in which number of contacts was the dependent variable. This uses negative binomial rather than poisson regression as the data were over-dispersed.

The pragmatic nature of the study, using the same data as would be available to PCOOH clinicians rather than a more detailed set of patient characteristics, adds additional constraints. The short length of consultation sequences reduces the accuracy of the statistical analysis and the absence of additional patient data means that other confounders could not be included. However, both these constraints are present in the actual delivery of urgent care services and so if the findings were to be useful they would need to handle this constraint.

### Relationship to other research

This is the first study, to our knowledge, which has attempted to measure complexity of reason for consultation in an urgent (out of hours) primary care setting with widely-used metrics from other disciplines. Recent studies from Norway] [27], Denmark [28] and Switzerland [29] have described the distribution of reason for encounter in out of hours primary care, however none have examined the sequence of reasons for encounter in individual patients. While some of the indices (e.g. Herfindal and Bice-Boxerman) have been used on short data sequences [14, 16, 23], others (e.g. Shannon entropy) were designed to use on longer sequences. Our aim in using this metric here was not to produce accurate values for individuals but rather to examine whether an informative signal could be detected in short noisy data sequences.

A number of authors have suggested that approaches derived from the science of complex systems have value in understanding healthcare – both qualitatively [30, 31] and quantitatively. In this study we have used the principle of measuring statistical complexity as a way of reproducibly describing consultation sequences as more or less complex.

### Implications for policy, practice and research

The first implication of our findings is that statistical complexity in reason for encounter does not increase with age. At first this appears unintuitive – as patients become older and develop more illnesses one should expect that the number and variety of reasons for encounter should increase. We propose two explanations for this finding. The first is that even where there is multi-morbidity, patients may have one dominant symptom which acts as a common pathway (for instance a patient with chronic lung disease may seek help for breathlessness even if the “trigger” is a respiratory infection or increased anxiety). The second is that patients may have a “natural threshold” for seeking help and whenever this is crossed – for whatever reason – it results in a contact. Some patients with mental health problems – particularly anxiety - may have lower thresholds [26]. The presence of high complexity in RfE, particularly in a younger adult, may be a useful indicator of concurrent anxiety.

In terms of predicting future contact, however, the complexity measures were uninformative. More predictive, was the presence of an explicit mental health problem in any of the previous consultation. Such problems are likely to include both severe mental illness and episodes of mental health crisis.

## Conclusion

Complexity of reason for encounter can be estimated in an out of hours primary care setting. While similar levels of complexity are seen in younger and older patients (suggesting that it is more to do with consultation behaviour than morbidity and may be a marker of health anxiety or somatoform disorder), it is not in itself a predictor of ongoing high use of urgent care.

## Additional files

Additional file 1: **Data 1.** Mapping of codes from original data to categories. (DOCX 16 kb)

Additional file 2: **Data 2.** Formulae for calculation of statistical measures of complexity. (DOCX 13 kb)

Additional file 3: **Figure S1.** Correlation plots of complexity measures (JPEG 356 kb)

Additional file 4: **Figure S2.** Complexity measures for individual patients with increasing number of contacts (limited to patients with > 20 contacts). (JPEG 832 kb)

Additional file 5: **Data 3.** Histograms of standardised residuals from regression models for different subgroups of patients. (DOCX 157 kb)

Additional file 6: **Figure S3.** Odds ratio for at least 33% more consultations based on predictors at the time of N^th^ consultation. (JPEG 414 kb)

Additional file 7: **Data 4** - Negative binomial regression with total number of contacts as the dependent variable. (DOCX 14 kb)

## Abbreviations

BDS: Bodily distress syndrome
ENT: Ear, Nose and Throat
ICPC-2: International Classification of Primary Care – 2nd Edition
PCOOH: Primary care out-of-hours
RfE: Reason for encounter
