# Supplementary material for: Statistical complexity of reasons for encounter in high users of out of hours primary care: analysis of a national service
Source: BMC Health Serv Res. 2019 Feb 8;19:108. doi: 10.1186/s12913-019-3938-z (PMC6368808; doi:10.1186/s12913-019-3938-z)
Supplement: Supplementary file 1 — Data 1. Mapping of codes from original data to categories. (DOCX 16 kb) [file 12913_2019_3938_MOESM1_ESM.docx]

**Accident**

Bites/stings

Bleeding

Burns

Falls

Foreign Bodies

ingestion/inhalation

Injury/wound

**Musculoskeletal**

Arms

Back

Buttock

Feet

Groin

Hand problems

Joints - ankle

Joints - elblow

Joints - hip

Joints - knee

Joints - shoulder

Joints - wrist

Legs

Neck

**Gastrointestinal**

Abdominal

Bowel problems

Constipation

Diarrhoea

Rectal/Anal

Vomiting/nausea

**Neurological / General**

Confusion

Dizziness

Face problems

Fainting

Fits

Headache

Head-related

Speech

Stroke

Weak

**Cardio-respiratory**

Breathing-related

Chest pain

Cough

Palpitations

**Mental health**

Drug abuse

Mental health

**Major Condition**

Cancer

Coughing/vomiting blood

Death

Diabetes

**Reproductive / urinary**

Breast problems

Genito-urinary

Male genitalia

Sexual health issues

Vaginal

**Skin/Eye/ENT**

Allergies

Dental problems

Ear problems

Eye problems

Lumps

Nose problems

Rash/Skin

Ulcers

**Upper Respiratory**

Fever

Flu or cold

Mouth

Throat problems

**Other**

Other miscellaneous

**Exclude: pregnancy / child**

Baby/Infant

Misc child/baby

Pregnancy-related

**Medication**

Medication

**Safety**

Safety questions

**Unclassified**

Not Available

Unclassified/unknown
