# Supplementary material for: Statistical complexity of reasons for encounter in high users of out of hours primary care: analysis of a national service
Source: BMC Health Serv Res. 2019 Feb 8;19:108. doi: 10.1186/s12913-019-3938-z (PMC6368808; doi:10.1186/s12913-019-3938-z)
Supplement: Supplementary file 2 — Data 2. Formulae for calculation of statistical measures of complexity. (DOCX 13 kb) [file 12913_2019_3938_MOESM2_ESM.docx]

Additional file 2**: Formulae used for measures of complexity of sequence.**

For a sequence of consultations including N different categories; and where P*_i_* represents the proportion of consultations belonging to the *i*^th^ category

$$Herfindahl index=\sum_{i=1}^{N} P_{i}^{2}$$

$$State Entropy=-\sum_{i=1}^{N} P_{i}*{log}_{2}\left( P_{i} \right)$$

If the same sequence is then converted to K different category transitions (so sequence ABBC becomes AB, BB, BC); and where P*_j_* represents the proportion of consultations belonging to the *j*^th^ transition category

$$Transition Entropy=-\sum_{j=1}^{K} P_{j}*{log}_{2}\left( P_{j} \right)$$
