# Supplementary figures and images for: Statistical complexity of reasons for encounter in high users of out of hours primary care: analysis of a national service
Source: BMC Health Serv Res. 2019 Feb 8;19:108. doi: 10.1186/s12913-019-3938-z (PMC6368808; doi:10.1186/s12913-019-3938-z)

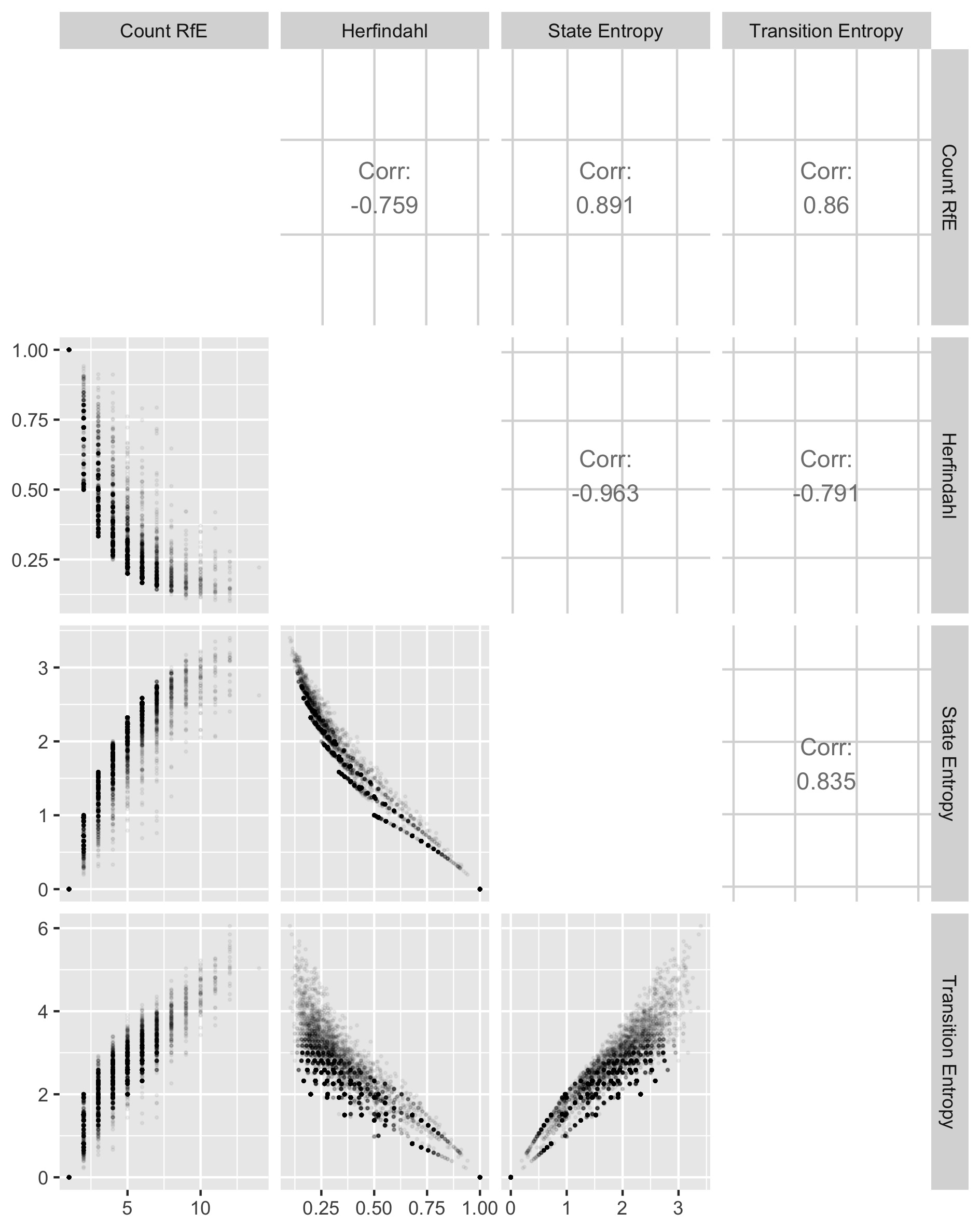

Supplement: Supplementary file 3 — Figure S1. Correlation plots of complexity measures (JPEG 356 kb) [file 12913_2019_3938_MOESM3_ESM.jpeg]

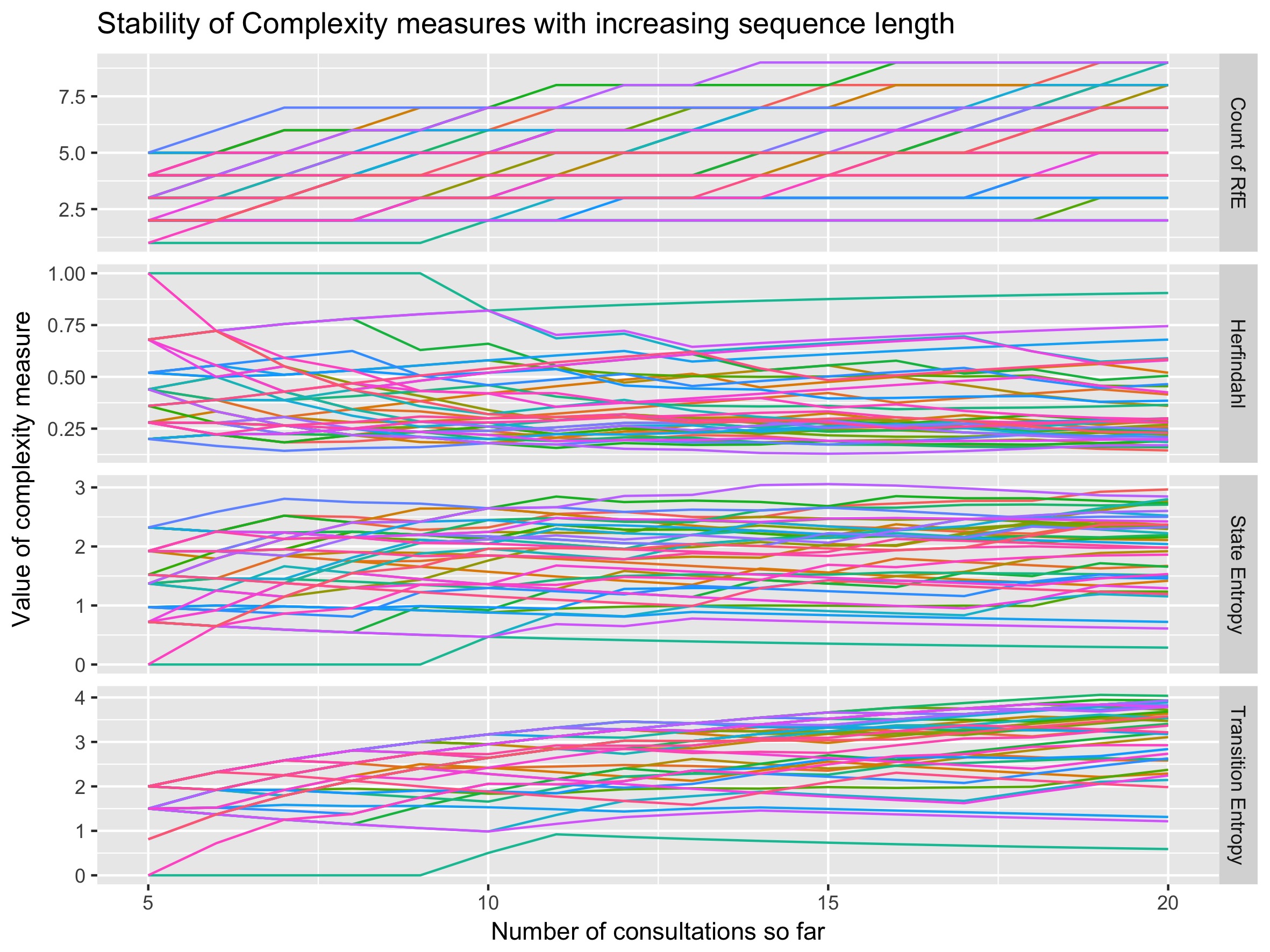

Supplement: Supplementary file 4 — Figure S2. Complexity measures for individual patients with increasing number of contacts (limited to patients with > 20 contacts). (JPEG 832 kb) [file 12913_2019_3938_MOESM4_ESM.jpeg]

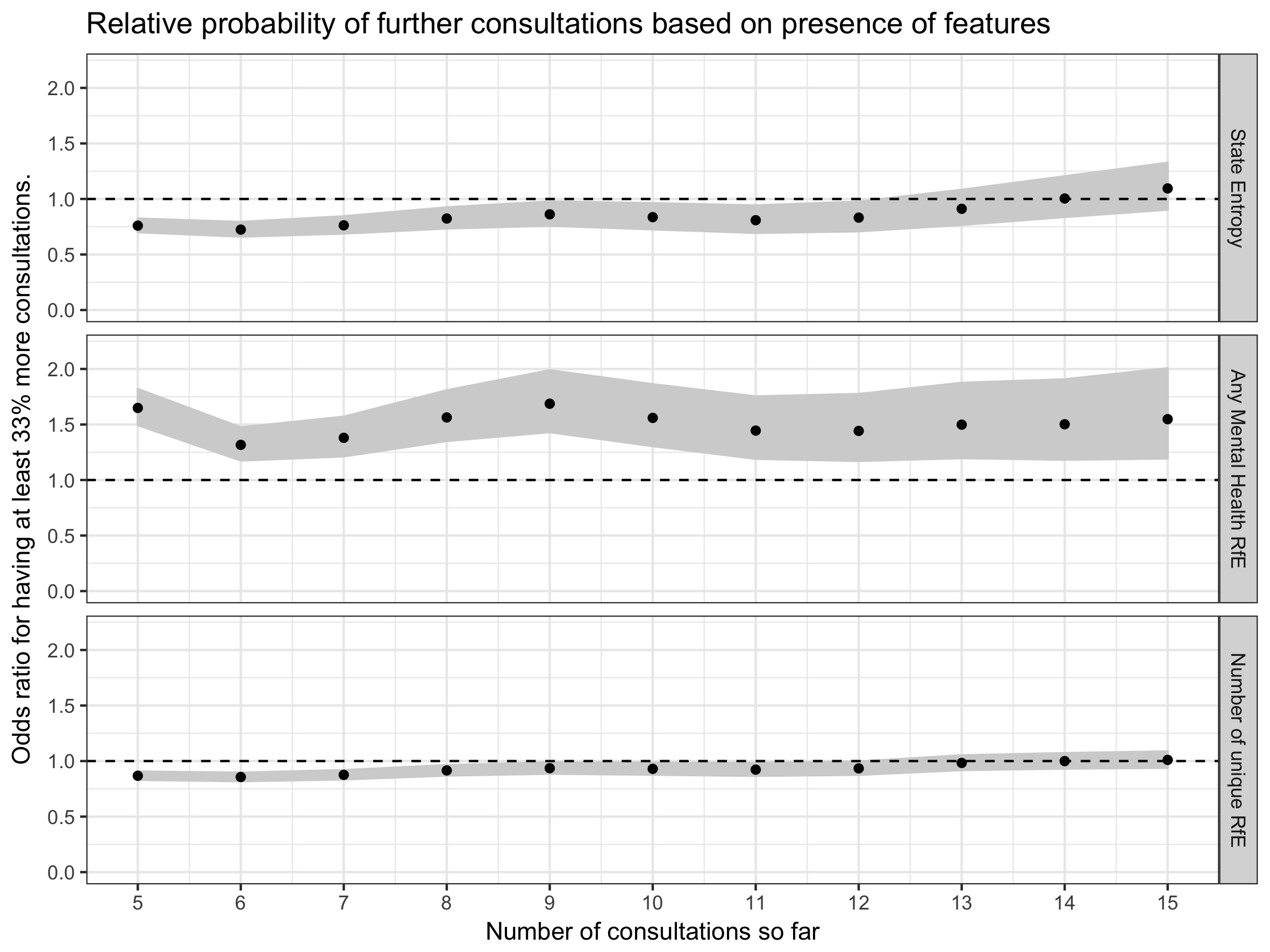

Supplement: Supplementary file 6 — Figure S3. Odds ratio for at least 33% more consultations based on predictors at the time of Nth consultation. (JPEG 414 kb) [file 12913_2019_3938_MOESM6_ESM.jpeg]
