# Supplementary material for: Statistical complexity of reasons for encounter in high users of out of hours primary care: analysis of a national service
Source: BMC Health Serv Res. 2019 Feb 8;19:108. doi: 10.1186/s12913-019-3938-z (PMC6368808; doi:10.1186/s12913-019-3938-z)
Supplement: Supplementary file 5 — Data 3. Histograms of standardised residuals from regression models for different subgroups of patients. (DOCX 157 kb) [file 12913_2019_3938_MOESM5_ESM.docx]

Additional file 5

**Histograms of standardised residuals from multiple regression analysis with all included patients and after excluding patients with >30 contacts.**

**
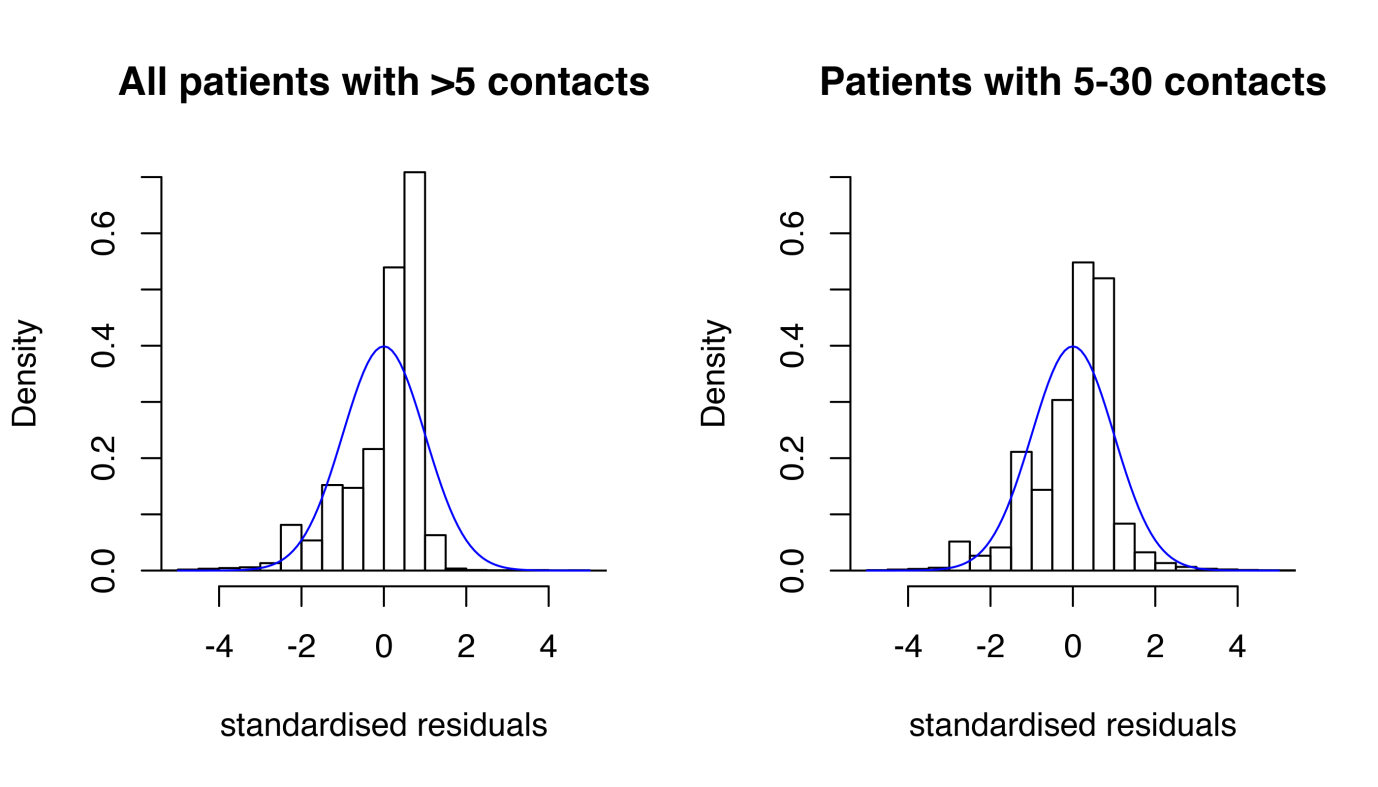
**

Blue line indicates normal distribution
