# Supplementary material for: Statistical complexity of reasons for encounter in high users of out of hours primary care: analysis of a national service
Source: BMC Health Serv Res. 2019 Feb 8;19:108. doi: 10.1186/s12913-019-3938-z (PMC6368808; doi:10.1186/s12913-019-3938-z)
Supplement: Supplementary file 7 — Data 4 - Negative binomial regression with total number of contacts as the dependent variable. (DOCX 14 kb) [file 12913_2019_3938_MOESM7_ESM.docx]

Additional file 7 – regression model for total number of contacts

Methods

We tested the association of variables with total number of contacts per individual using negative binomial regression because of the heavy tailed distribution which showed over-dispersion. Analysis was carried out on a dataset including all patients. We included the same variables as for the main regression in the model (Shannon entropy, patient age and sex and whether any consultation was for a mental health problem) and ran models including and excluding the number of RfE categories. We reported the results as Incidence Rate Ratio: the exponentiated coefficient for each variable with 95% confidence intervals.

Results

Number of RfE categories was the strongest predictor of total number of contacts as shown in Supplementary Table 5. To some extent this is unsurprising, as a high number of categories required a high number of contacts. The IRR <1 for Shannon entropy in supplementary table 5 is at first sight unintuitive, but it is only seen in the presence of the number of RfE categories variable (when that is removed the IRR for Shannon entropy is 1.3) indicating that higher complexity is associated with total contact count. However when controlling for the number of categories it appears that higher entropy is associated with lower total numbers of contacts.

Supplementary Table 5: Results of negative binomial regression with total number of contacts as the dependent variable.

|  | IRR | 95%CI | p-value |
| --- | --- | --- | --- |
| Number of RfE categories | 3.74 | 3.64 to 3.83 | <.001 |
| Shannon entropy | 0.07 | 0.07 to 0.08 | <.001 |
| Age (decades) | 0.99 | 0.98 to 1.0 | 0.04 |
| Sex: Male | 1.00 | 0.96 to 1.04 | 0.98 |
| Mental Health RfE | 1.07 | 1.02 to 1.13 | 0.009 |
|  |  |  |  |

IRR – incidence rate ratio

RfE Reason for Encounter
